# Concurrent Chylothorax, Chylopericardium, and Superior Vena Cava Syndrome in a Young Male With Primary Mediastinal Large B-cell Lymphoma: A Report of a Rare Case

**DOI:** 10.7759/cureus.93881

**Published:** 2025-10-05

**Authors:** Sanu Lama, Shivam Singla, Bhavna Singla, Albina Mercy, Rana Salman

**Affiliations:** 1 Research, Prime Healthcare, Ontario, USA; 2 Internal Medicine, Janaki Medical College and Teaching Hospital, Janakpur, NPL; 3 Internal Medicine, TidalHealth Peninsula Regional, Salisbury, USA; 4 Internal Medicine, Erie County Medical Center Health Campus, Buffalo, USA; 5 Internal Medicine, Davao Medical School Foundation, Inc., Davao, PHL; 6 Internal Medicine, Bahawal Victoria Hospital, Bahawalpur, PAK

**Keywords:** chylopericardium, chylothorax, da-epoch-r, mediastinal mass, primary mediastinal large b-cell lymphoma, superior vena cava syndrome

## Abstract

Primary mediastinal large B-cell lymphoma (PMBCL) is an uncommon and aggressive subtype of non-Hodgkin lymphoma (NHL) that predominantly affects young adults and typically presents with a bulky anterior mediastinal mass. We report the case of a 23-year-old male who presented with progressive dyspnea, orthopnea, and facial swelling, subsequently found to have superior vena cava (SVC) syndrome, chylothorax, and chylopericardium, an exceedingly rare triad of complications. Diagnostic evaluation required integration of advanced imaging, fluid analysis, and histopathological confirmation, ultimately establishing a diagnosis of stage III PMBCL with low-level bone marrow involvement. The patient was initiated on dose-adjusted rituximab, etoposide, prednisone, vincristine, cyclophosphamide, and doxorubicin (DA-EPOCH-R) chemotherapy, with early symptomatic improvement during the first cycle. This case highlights the diagnostic challenges and management complexities of atypical PMBCL presentations and underscores the importance of early recognition and prompt initiation of systemic therapy in patients presenting with recurrent chylous effusions and mediastinal pathology.

## Introduction

Primary mediastinal large B-cell lymphoma (PMBCL) is a distinct clinicopathologic entity that accounts for a small proportion of non-Hodgkin lymphoma (NHL) and diffuse large B-cell lymphoma (DLBCL). It most often arises from thymic B-cells in young adults, typically in the third to fourth decade of life, and shows a slight female predominance [1]. Clinically, PMBCL presents as a rapidly enlarging anterior mediastinal mass that may cause compressive symptoms such as superior vena cava (SVC) syndrome, airway obstruction, and pericardial effusion [2].

Histopathologic confirmation relies on identifying large atypical B-cells expressing markers such as cluster of differentiation 20 (CD20), cluster of differentiation 79a (CD79a), and paired box protein 5 (PAX5), often with variable cluster of differentiation 30 (CD30) and B-cell lymphoma 6 protein (BCL6) expression. Standard treatment involves rituximab-based multi-agent chemotherapy, with dose-adjusted rituximab, etoposide, prednisone, vincristine, cyclophosphamide, and doxorubicin (DA-EPOCH-R), demonstrating excellent long-term outcomes and frequently eliminating the need for consolidative radiotherapy. Despite its overall favorable prognosis, a proportion of patients experience relapsed or refractory disease, requiring advanced therapies including checkpoint inhibitors and chimeric antigen receptor T-cell (CAR-T) therapy [3].

Although pericardial effusion and SVC syndrome are recognized complications, the simultaneous occurrence of chylopericardium, chylothorax, and SVC obstruction is exceedingly rare and poses significant diagnostic and therapeutic challenges. In this report, we present the case of a 23-year-old male with stage III PMBCL complicated by pericardial effusion, SVC syndrome, and concurrent chylothorax and chylopericardium, an exceptionally rare constellation of findings. The aim is to highlight the complexity of such presentations, discuss the diagnostic approach, and review management strategies within the context of current evidence.

## Case presentation

A 23-year-old previously healthy male presented to the emergency department with progressive shortness of breath, orthopnea, and swelling of the face. His symptoms began six weeks earlier with exertional dyspnea, which gradually worsened to the point where he became breathless even after walking a few steps and experienced significant discomfort on lying flat. Approximately one week after symptom onset, he developed facial swelling that started periorbitally and then progressed to involve the entire face. He initially sought care at a local hospital, where baseline laboratory investigations were unremarkable. A chest radiograph, however, revealed cardiomegaly with a characteristic “water-bottle” configuration highly suggestive of a large pericardial effusion, along with possible mediastinal widening concerning for either a mass lesion or vascular congestion.

He was subsequently referred to a cardiac specialty center, where echocardiography confirmed a large pericardial effusion with early diastolic right ventricular collapse, consistent with impending tamponade. Immediate pericardiocentesis was performed, and a pericardial drain was left in place for one week, yielding approximately two liters of fluid. Cytological examination of the fluid demonstrated reactive mesothelial cells, lymphocytes, and neutrophils without malignant cells. The patient experienced transient improvement and was discharged; however, he returned one week later to a tertiary care hospital with recurrence of dyspnea, orthopnea, and facial swelling.

On admission, laboratory evaluation, including complete blood count, renal and liver function, coagulation profile, and inflammatory markers, revealed no major abnormalities apart from a mild elevation in C-reactive protein and lactate dehydrogenase. The baseline investigations are summarized in Table 1.

**Table 1:** Baseline laboratory investigations on admission. WBC: white blood cell; LDH: lactate dehydrogenase; ESR: erythrocyte sedimentation rate; CRP: C-reactive protein; ALT: alanine aminotransferase; AST: aspartate aminotransferase

Parameter	Result	Reference Range
Hemoglobin	13.3 g/dL	13–18 g/dL
WBC count	8.4 ×10⁹/L	4–11 ×10⁹/L
Platelets	234 ×10⁹/L	150–400 ×10⁹/L
LDH	330 U/L	140–280 U/L
ESR	20 mm/hr	0–25 mm/hr
CRP	30 mg/L	<5 mg/L
Serum creatinine	0.9 mg/dL	0.5–0.9 mg/dL
ALT/AST	30/38 U/L	<40 U/L

**Figure 1:**
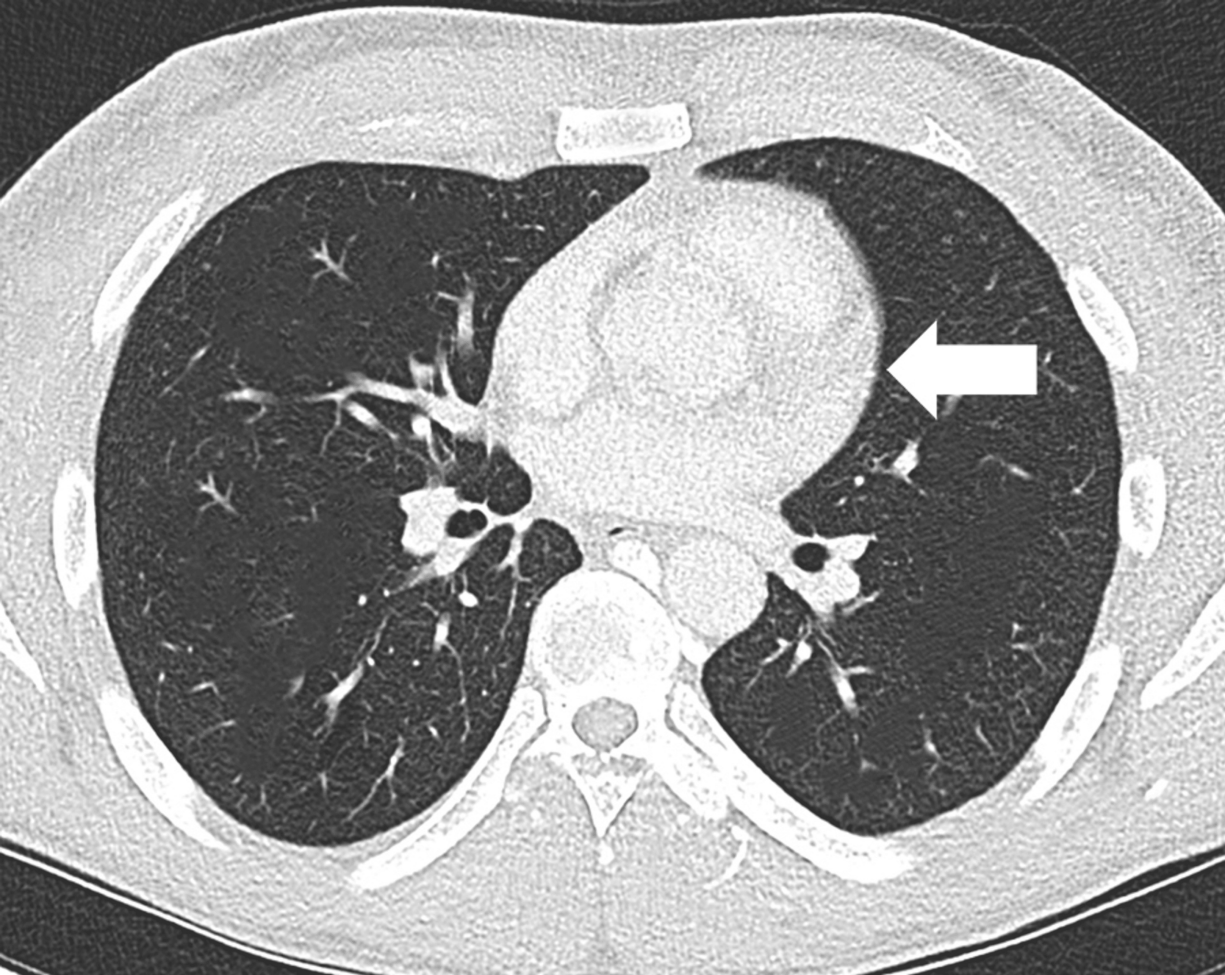
HRCT represents a large soft tissue mass in the anterior mediastinum, as pointed by the arrow. The mass appears to compress the superior vena cava. HRCT: high-resolution computed tomography

High-resolution computed tomography (HRCT) of the chest, as given in Figure 1, demonstrated a large anterior mediastinal mass compressing and partially encasing the SVC, correlating with the patient’s facial swelling and orthopnea. The mass displaced the heart and was associated with mild right pleural effusion, mediastinal lymphadenopathy, and patchy adjacent atelectasis, but no large recurrent pericardial effusion. A right-sided chest tube was placed for drainage of the pleural effusion, which yielded 1,000 mL of milky fluid within 12 hours.

A homogenous opacification is noted in the right lower zone, with the opacity seen to track along the lateral chest wall. The right costophrenic angle is obliterated, with a meniscus noted. Findings are suggestive of a right-sided pleural effusion.

Chemical analysis of the pericardial and pleural fluids revealed elevated triglyceride levels (376 mg/dL in pericardial fluid; 215 mg/dL in pleural fluid) with lymphocytic predominance, establishing the diagnosis of chylopericardium and chylothorax. Given the absence of recent surgery or trauma, malignancy was strongly suspected as the underlying cause of his recurrent effusions.

**Figure 2:**
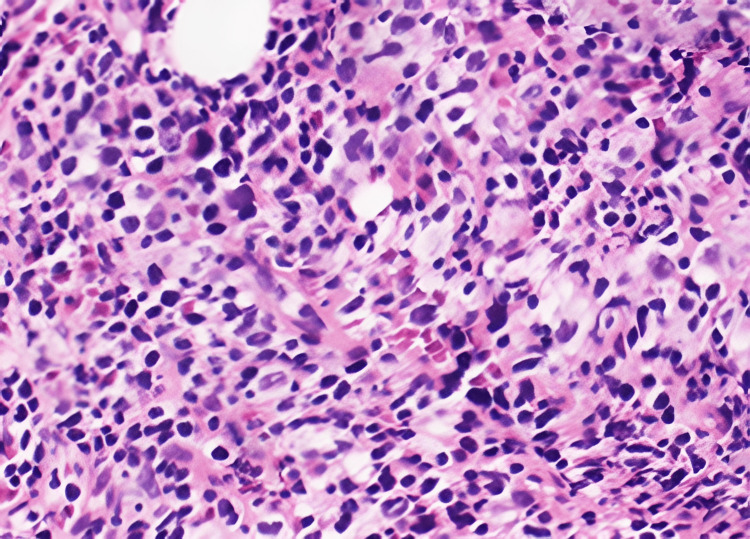
Bone marrow biopsy revealed normocellular marrow with preserved hematopoiesis but focal clusters of atypical large lymphoid cells.

Supportive management was initiated with supplemental oxygen, intravenous dexamethasone to reduce mediastinal edema, head elevation to relieve venous congestion, and cautious intravenous hydration. Further diagnostic work-up was pursued. Bone marrow biopsy revealed normocellular marrow with preserved hematopoiesis but focal clusters of atypical large lymphoid cells, as illustrated by Figure 2, expressing CD20 and PAX5 with a proliferative index of approximately 60%, indicating low-level marrow involvement. Core needle biopsy of the mediastinal mass demonstrated diffuse sheets of large atypical lymphoid cells with vesicular chromatin, prominent nucleoli, and abundant pale cytoplasm arranged in a fibrosclerotic background. Immunohistochemistry showed strong diffuse positivity for CD20, CD79a, PAX5, BCL6, and multiple myeloma oncogene 1 (MUM1), with variable CD30 expression and a high Ki-67 index (~70%), confirming the diagnosis of PMBCL.

A staging positron emission tomography-computed tomography (PET-CT) scan revealed a large fluorodeoxyglucose (FDG)-avid anterior mediastinal mass with a maximum standardized uptake value (SUV) of 18.2, along with FDG-avid supraclavicular, mediastinal, axillary, retroperitoneal, and para-aortic lymph nodes, but no visceral organ involvement. Mild non-FDG-avid pericardial effusion persisted, while the bone marrow biopsy findings were consistent with low-level infiltration. Overall, the disease was staged as Ann Arbor stage III with a Deauville score of 5.

The patient was commenced on DA-EPOCH-R chemotherapy. The regimen consisted of continuous infusion of etoposide, doxorubicin, and vincristine over four days; cyclophosphamide on day five; rituximab on day one; and prednisone orally for five days. Supportive care included granulocyte colony-stimulating factor (G-CSF) from day six until neutrophil recovery, tumor lysis prophylaxis with allopurinol, and antiemetics. A central venous catheter (CVC) was inserted for chemotherapy administration.

By day 15 of the first cycle, the patient demonstrated marked improvement in dyspnea and resolution of facial swelling, with stable hemodynamic and laboratory parameters. He was planned to complete six cycles of DA-EPOCH-R, with interim PET-CT after two to four cycles to assess treatment response, followed by end-of-treatment PET-CT to confirm remission. If a complete metabolic response is achieved, consolidative radiotherapy will not be required. Given his young age, good performance status, and early symptomatic improvement, his prognosis is considered favorable, with expected long-term remission rates exceeding 80% in similar patients.

## Discussion

PMBCL is a rare but aggressive subtype of NHL, typically affecting young adults. It arises from thymic B-cells and most often manifests as a bulky mediastinal mass, with compressive complications such as SVC syndrome, pericardial effusion, and airway compromise being well-documented [4]. Our case illustrates not only these classic complications but also the unusual coexistence of chylopericardium and chylothorax, representing a highly uncommon constellation of findings that underscores both the biological aggressiveness of the disease and the diagnostic challenges it poses.

The pathophysiology of PMBCL-related complications is primarily related to the direct mass effect. Involvement of the SVC can cause venous obstruction, leading to facial swelling, upper limb edema, and orthopnea, as observed in our patient [5]. Pericardial effusion occurs in up to one-third of patients, often secondary to pericardial infiltration or obstruction of pericardial lymphatic drainage. Cardiac tamponade is a life-threatening complication and requires prompt intervention.

What distinguishes our case from typical presentations is the simultaneous development of chylothorax and chylopericardium, which are rarely reported in PMBCL. Chylous effusions arise due to obstruction or disruption of the thoracic duct or its tributaries, leading to leakage of lymph rich in triglycerides into the pleural or pericardial space. In malignant cases, mediastinal masses compressing the thoracic duct are a well-known mechanism, but reports specifically linking PMBCL to concurrent chylothorax and chylopericardium are extremely limited [6]. This combination complicates management, as recurrent fluid collections can mimic infection or pericarditis, delay diagnosis, and compromise cardiorespiratory function.

In our patient, the initial cytology of pericardial fluid was negative for malignant cells, highlighting a common diagnostic pitfall. Reactive mesothelial cells and lymphocytic predominance can obscure an underlying malignant etiology. Reliance solely on fluid cytology may therefore provide false reassurance, delaying definitive diagnosis. This underscores the importance of integrating cross-sectional imaging, fluid chemistry, and tissue biopsy when evaluating young patients with recurrent chylous effusions [7]. Notably, the elevated triglyceride levels in both pericardial and pleural fluids were key to establishing the chylous nature of the effusions, which in turn prompted a search for malignancy.

A second diagnostic challenge lies in differentiating PMBCL from other anterior mediastinal malignancies, such as classical Hodgkin lymphoma, thymic carcinoma, and mediastinal germ cell tumors. The histopathologic profile in our case, with CD20, PAX5, and CD79a positivity, and variable CD30 expression in a fibrosclerotic stroma, was typical of PMBCL and helped exclude close differentials [8]. This reinforces the importance of immunohistochemistry in accurately classifying mediastinal masses, as treatment regimens and prognosis differ significantly across entities.

Treatment of PMBCL has evolved from CHOP-based regimens to rituximab-containing, dose-intensified protocols. DA-EPOCH-R is now widely accepted as the preferred frontline regimen, with studies demonstrating survival rates exceeding 80% without the need for routine consolidative radiotherapy [9]. In our patient, initiation of DA-EPOCH-R resulted in rapid clinical improvement and stabilization after the first cycle, consistent with the robust chemosensitivity reported in the literature.

From a supportive standpoint, management of malignant chylothorax and chylopericardium remains a therapeutic challenge. Initial measures such as pericardiocentesis and chest tube drainage are necessary for stabilization but are rarely curative if the underlying obstruction persists. Dietary modification (medium-chain triglycerides, total parenteral nutrition) and somatostatin analogs have been used in selected cases to reduce chyle flow, though data are limited in lymphoma-related effusions. In our case, definitive control of effusions was achieved only after systemic chemotherapy reduced tumor burden, underscoring that disease-directed therapy remains the cornerstone of managing malignant chylous effusions.

Reports of PMBCL presenting with both chylothorax and chylopericardium in the setting of SVC syndrome are extremely scarce. Most of the available literature discusses these complications in isolation, either as secondary to thoracic duct disruption following trauma or surgery or as rare sequelae of lymphoproliferative disorders [10]. Our case highlights an unusual clinical scenario in which a single disease process produced three life-threatening complications simultaneously. This observation provides several important clinical insights. First, young patients presenting with recurrent chylous effusions should be carefully evaluated for mediastinal malignancies, even when initial cytology is negative. Second, early integration of advanced imaging and tissue biopsy is critical to avoid diagnostic delays and misclassification. Finally, in cases of malignant chylous effusions, definitive resolution is rarely achieved with drainage alone and almost always requires effective systemic therapy directed at the underlying malignancy [11].

Although this case contributes valuable insights into the rare and complex presentations of PMBCL, a key limitation lies in the unavailability of high-quality, publishable versions of certain diagnostic images, including the chest radiograph, echocardiogram, and PET-CT. Despite repeated efforts, institutional archiving and equipment constraints prevented us from retrieving these figures in adequate resolution. Nevertheless, we were able to include HRCT and histopathological images, and the detailed clinical, laboratory, and management data presented provide sufficient diagnostic clarity and educational value. Future reports from similar settings would benefit from the availability of comprehensive imaging and pathology figures to further enhance understanding of such uncommon clinical scenarios.

## Conclusions

This case underscores the rare and complex presentation of PMBCL in a young male, complicated by the simultaneous occurrence of chylothorax, chylopericardium, and SVC syndrome, an exceptionally uncommon constellation of life-threatening complications. The diagnostic pathway required a comprehensive multimodal approach, combining advanced imaging, fluid analysis, and histopathology to establish the diagnosis and guide management. The patient’s early improvement with DA-EPOCH-R highlights the importance of prompt systemic therapy in malignant chylous effusions, where drainage alone is insufficient. This report expands the clinical spectrum of PMBCL presentations and reinforces the need for early suspicion of mediastinal malignancy in young patients with recurrent chylous effusions, offering valuable insight for timely recognition and intervention in future cases.
